# 

**Published:** 1909-05-15

**Authors:** 


### Dental Register

LIBRARY
Indexed
S. G. O.

NELVILLE S. HOFF, Editor,
Ann Arbor, Mich.

Vol. LXIII — MAY, 1909 — No. 5

SAMUEL A. CROCKER & CO., Publishers
OHIO DENTAL AND SURGICAL DEPOT
35 W. FIFTH STREET, CINCINNATI, O.

PRICE, ONE DOLLAR A YEAR

Entered at the Postoffice at Cincinnati, O, as second-class mail matter

